# The effect of information provision on consumers’ risk perceptions of, support for a ban, and behavioral intention towards the preventive use of antibiotics in food animals

**DOI:** 10.1186/s12889-024-18859-2

**Published:** 2024-05-28

**Authors:** Yingnan Zhou, Airong Zhang, Rieks Dekker van Klinken, Junxiu Wang

**Affiliations:** 1 Health and Biosecurity, Commonwealth Scientific and Industrial Research Organisation (CSIRO), Brisbane, QLD 4102 Australia; 2 School of Sociology and Ethnology, University of Chinese Academy of Social Sciences, Beijing, 102488 China; 3 School of Mental Health, Wenzhou Medical University, Wenzhou, Zhejiang 325035 China; 4 The Affiliated Kangning Hospital of Wenzhou Medical University, Wenzhou, Zhejiang 325007 China

**Keywords:** Information provision, Risk perception, Support for a ban, Behavioral intention, The preventive use of antibiotics, Food animals

## Abstract

**Background:**

Antibiotics have been widely used in feed and drinking water for food animals to prevent them from getting sick. Such preventive use of antibiotics has become a contributor to increasing antibiotic resistance and thus poses threats to human health. However, consumers have little knowledge about this practice and the associated health risks of increasing transmission of antibiotic residues and antibiotic resistant bacteria. This study aimed to examine the effect of information provision on consumers’ risk perceptions, support for a ban, and behavioral intention regarding the preventive use of antibiotics in food animals. Especially, the study sought to test two competing hypotheses which were informed by two theoretical perspectives of fear appeal theory — the linear model and the plateau effect model. The former suggested that providing information on the health risks of both antibiotic residues and antibiotic resistant bacteria would have a stronger effect compared to providing information on only one of them, while the latter posited that providing information on both risks might not have additional influence, as the effect of information on either risk could reach the plateau.

**Methods:**

An experimental study with four conditions was conducted where participants read different information on the health risks associated with the preventive use first and then answered questions regarding consumers’ risk perceptions, support for a ban, and behavioral intention regarding the preventive use. Condition 1 was the control condition, where basic information about antibiotics, antibiotic resistance, and the preventive use was provided. Condition 2 and Condition 3 further added information on the health risk of antibiotic residues (Condition 2) and antibiotic resistant bacteria (Condition 3) due to the preventive use, respectively. Condition 4 provided all information contained in the first three conditions.

**Results:**

The results showed that compared to participants in the control condition, participants in Conditions 2-4 reported higher risk perceptions, stronger support for a ban on the preventive use, and a higher intention to buy meat produced without the preventive use of antibiotics. However, there were no significant differences in these factors between Conditions 2-4, indicating that providing information on the health risk of either antibiotic residues, or antibiotic resistant bacteria, or both, has similar effect on these variables. That is, the hypothesis based on the plateau effect model was supported.

**Conclusions:**

The findings suggested that informing the public with the health risk of either antibiotic residues or antibiotic resistant bacteria associated with the preventive use is effective enough to reach plateau effect in increasing risk perceptions, support for a ban, and behavioral intention, which has important implications for policymakers and livestock industries to develop effective communication strategies to promote responsible antibiotic use in food animals.

## Background

Antibiotic resistance has posed a serious health threat worldwide. New antibiotic resistant bacteria are emerging and spreading globally, leading to increased mortality and higher health care burden [1]. It was estimated that antibiotic resistant infections were responsible for 1.27 million deaths in 2019 [2]. Without urgent action, the figure is projected to reach 10 million deaths every year by 2050 [1]. Moreover, antibiotic resistant bacteria are spreading within and between different ecosystems, thus has become a global ecological problem affecting the health of humans, animals, and the environment [3, 4]. Antibiotic resistance is largely accelerated by the inappropriate use and overuse of antibiotics in multiple sectors, especially in humans and food animals [5]. The use of antibiotics and the action taken to control antibiotic resistance in one sector affects the others [5, 6]. Therefore, to address the health threat posed by increasing antibiotic resistance, it’s necessary to take a One Health approach [3, 7, 8]. One Health is an integrated and unifying approach that mobilizes collaborative effort of multiple sectors, disciplines, and communities to sustainably balance and optimize the health of humans, animals, and the environment [9]. From One Health perspective, it’s important to reduce the inappropriate use of antibiotics in both humans and food animals. Although a growing body of evidence has demonstrated the link between antibiotic use in food animals and increasing antibiotic resistance [10–13], compared to antibiotic misuse and antibiotic resistance in humans, inappropriate use of antibiotics and antibiotic resistance in food animals has drawn less attention [14].

Antibiotics administered to food animals include therapeutic use and subtherapeutic use. Therapeutic use refers to using antibiotics to treat infectious diseases in sick animals [15]. The therapeutic use of antibiotics is essential for treating bacterial infections and protecting animal welfare. Subtherapeutic use refers to administering antibiotics to animals with doses lower than therapeutic use for a longer period to prevent healthy animals from getting sick or to promote growth [16–18]. In this case, antibiotics are usually added to feed or water [19, 20]. Recently, many countries (e.g., the EU, the US, and China) have banned antibiotics from being used as growth promoters [21–23].

The preventive use of antibiotics was, however, only banned in the EU countries since 2022 and is still allowed in most countries globally [19, 24]. Farmers routinely added antibiotics in feed or water to prevent diseases in groups of animals in various countries such as Brazil [25], Cambodia [26], Thailand [27], Vietnam [28], and China [29]. In Thailand, an average of 303 mg of antibiotics including tilmicosin, doxycycline, amoxicillin, colistin, and oxytetracycline were given to each chicken for disease prevention during the 41 days of raising period [27]. In Brazil, antibiotics were widely used for disease prevention among sows, newborn piglets, and weaning pigs [25]. Farmers alternately used antibiotics of different classes (e.g., aminopenicillin, pleuromutilin, amphenicol, polymyxin, tetracycline, quinolone, and macrolide) to prevent diseases in piglets. As a result, a piglet could be exposed to more than five antibiotic classes between 28 and 70 days of life [25]. Such preventative use of antibiotics in healthy animals may have serious consequences for human health [5, 30]. Therefore, the present study focused on the preventive use of antibiotics in food animals.

### The health risk associated with *antibiotic residues* due to the preventive use of antibiotics in food animals

The preventive use of antibiotics in food animals may result in antibiotic residues presenting in food [16, 31, 32]. Antibiotic residues have been found in various animal-derived food, such as meat, fish, milk, and eggs in many countries and regions globally (e.g., the US, Brazil, Cameroon, Egypt, Ghana, Greece, Nigeria, Bangladesh, Zambia, Iran, Kenya, China, India, and South Africa) [32, 33]. High concentration of antibiotic residues in food can have direct toxic effects on human beings [16, 31, 32]. Among those, allergic reactions against *β*-lactam antibiotic (e.g., cephalosporin and penicillin) residues in meat or milk are most common [6]. The symptoms may include acute interstitial nephritis, vasculitis, skin rashes, bronchospasm, acute interstitial nephritis, vasculitis, serum sickness, erythema multiforme, toxic epidermal necrolysis, hemolytic anemia, anaphylaxis thrombocytopenia, angioedema, and Stevens–Johnson syndrome [6, 34]. Antibiotic residues in food may also damage the immune system, organs (i.e., liver, kidneys, and reproductive organs), and bone marrow, as well as increase the chance of mutations and carcinoma [31, 35, 36].

### The health risk associated with *antibiotic resistant bacteria* due to the preventive use of antibiotics in food animals

The preventive use of antibiotics in food animals has become a great contributor to the increase of antibiotic resistant bacteria both in animals and in the environment, thus increases the risk of humans getting infected with antibiotic resistant bacteria [37–40]. Studies have found a strong association between the prevalence of antibiotic resistant bacteria in food animals and in human beings [41, 42]. The most common antibiotic resistant foodborne bacteria affecting human health are antibiotic resistant E. coli, salmonella, campylobacters, and enterococci [16, 43]. A growing body of evidence has shown an increasing prevalence of these antibiotic resistant bacteria in animals. For instance, Roth et al. investigated the prevalence of antibiotic resistant bacteria in poultry in the US, China, Brazil, and the EU. They found the average proportion of antibiotic resistant E. coli isolated from chickens was over 40% [44]. Van Boeckel et al. revealed that the prevalence of antibiotic resistant bacteria (i.e., E. coli, campylobacters, salmonella, and staphylococcus aureus) in chickens, pigs, and cattle has all largely increased from 2000 to 2018 in low- and middle-income countries [14]. Furthermore, animals cannot fully metabolize the administered antibiotics. Consequently, antibiotic residues and antibiotic resistant bacteria are discharged into the environment (i.e., water and soils) through manure [20, 45, 46]. High level of antibiotic resistant bacteria has been found in almost all parts of the environment, including soil [47, 48], freshwater aquaculture ponds [49], rivers [50], groundwater [51], sediments and sea water [52]. Consequently, humans may get infected with antibiotic resistant bacteria when they have direct contact with animals, handle contaminated food, consume undercooked food, consume contaminated water and vegetables, or have contact with antibiotic resistant bacteria in the environment [4, 53]. If humans are infected with antibiotic resistant bacteria, it would be more difficult, or even impossible to treat as existing antibiotics have become ineffective in treating these bacterial infections [1]. For instance, antibiotic resistant foodborne bacteria E. coli and Staphylococcus aureus infections were responsible for over 500, 000 deaths in 2019 [2].

### Consumers’ knowledge about, perceptions of, and attitudes towards the preventive use of antibiotics in food animals

Research so far has mainly focused on investigating consumers’ knowledge about, perceptions of, and attitudes towards the overall use of antibiotics in food animals [54–65]. The results of these studies suggested that, although having little knowledge about antibiotic use in food animals and its contribution to antibiotic resistance, consumers somehow believed antibiotics are widely used in livestock industries and concerned about it [54–60, 62, 63]. Moreover, the public concern about overall antibiotic use in food animals has facilitated the purchase demand and higher willingness to pay for animal-derived food produced without any use of antibiotics [66, 67]. In response to this consumer demand, “antibiotic-free” or “raised without antibiotics” labelled food products have emerged in many countries (e.g., the US, Germany, the UK, Italy, and Australia) [55, 56, 68, 69]. However, removing therapeutic use of antibiotics in livestock is detrimental to both animal welfare and food safety [55, 70]. Therefore, from the perspective of One Health, it’s of extreme importance to promote responsible antibiotic use in food animals rather than eliminating antibiotics altogether, as it can balance and optimize the health of humans, animals, and the environment. The purchase demand for antibiotic-free animal-derived food might be due to concern about food safety and preference for less chemicals and additives in food [64, 71, 72], the lack of knowledge about the difference between therapeutic and subtherapeutic use of antibiotics, and the misunderstanding that all antibiotics used in food animals are harmful [73].

Hence, it’s necessary to differentiate the preventative use from therapeutic use of antibiotics, and to investigate consumers’ knowledge about, perceptions of, and attitudes towards the preventive use of antibiotics separately. However, only limited studies have shed some light on this area. Research in Ireland found most consumers were unfamiliar with the preventive use of antibiotics in food animals and were surprised when being informed about it, because they thought that antibiotics can only be used for treatments [62]. On the other hand, some consumers considered the preventive use of antibiotics as a normative and standard practice in farming [62]. Research in the US revealed that only about one third of consumers were very concerned about the preventive use of antibiotics in food animals and even less of them considered the preventive use as unacceptable [61]. These findings suggested that consumers have little knowledge about the preventive use of antibiotics in food animals and limited understanding of the health risks associated with it.

Emerging experimental studies suggested that receiving information on the health risk of antibiotic resistance associated with antibiotic use in food animals could significantly increase consumers’ knowledge and risk perception regarding antibiotic use and antibiotic resistance in food animals [60, 74] as well as willingness to pay for antibiotic-free animal-derived food [66, 67]. However, some of these studies focused on the hazard of using antibiotics for promoting growth [66]. While others focused on the health risk in relation to the overall use of antibiotics in food animals without differentiating the preventative use and therapeutic use [60, 67, 74].

### The present study

Given that consumers have little knowledge about the preventive use of antibiotics in food animals (hereafter referred to as “the preventive use”), this study sought to improve consumers’ knowledge through providing information on the health risks associated with the preventive use, and investigating its effect on risk perceptions, support for a ban, and behavioral intention. The current research applied quasi-experimental methodology [75, 76] to systematically present information on the health risks associated with the preventive use. This allowed us to explore if variations in information can affect consumers’ risk perceptions, support for a ban, and behavioral intention regarding the preventive use.

We anticipated that increased knowledge about the health risks of antibiotic residues and antibiotic resistant bacteria associated with the preventive use would influence risk perceptions, support for a ban, and behavioral intention towards the preventive use. We could not assume whether there would be significant differences in the effects of increased knowledge on antibiotic residues only versus knowledge on antibiotic resistant bacteria only, as there is no existing literature allowing us to make any assumptions.

Further, we hypothesized that, compared to providing knowledge on either antibiotic residues only or antibiotic resistant bacteria only, providing knowledge on both antibiotic residues and antibiotic resistant bacteria associated with the preventive use would either lead to a stronger effect on changes in risk perceptions, support for a ban, and behavioral intention, or result in no further increase. These competing hypotheses were informed by research based on fear appeal theory. Fear appeal is a persuasive communication strategy aiming at promoting attitude and behavioral changes by arousing fear via emphasizing the potential risk [77, 78]. Research has suggested two theoretical perspectives of fear appear — the linear model and the plateau effect model. The linear model posits a positive linear relationship between depicted fear and persuasion, such that the more fear depicts, the more effective the information is in affecting risk perceptions, attitudes, and behavioral intentions [79–83]. From the perspective of the linear model, providing information about the health risks of both antibiotic residues and antibiotic resistant bacteria would have a stronger effect compared to providing information on only one of them. On the other hand, the plateau effect model suggests that the effect of depicted fear will reach a plateau at certain point, beyond which depicting additional fear has no additional influence on risk perceptions, attitudes, intentions, and behaviors [78, 84]. From this perspective, providing information on both antibiotic residues and antibiotic resistant bacteria may not have additional influence, as it is likely that the effect of increased knowledge on either antibiotic residues only or antibiotic resistant bacteria only would reach its plateau.

Taken together, we hypothesized that providing information on the health risk of either antibiotic residues, or antibiotic resistant bacteria, or both caused by the preventive use would significantly increase consumers’ risk perceptions, support for a ban, and behavioral intention regarding the preventive use. We further hypothesized that providing information on the health risks of both antibiotic residues and antibiotic resistant bacteria would have an either stronger or similar effect on these variables in comparison with providing information on only one of them.

## Methods

### Research design

**Fig. 1:**
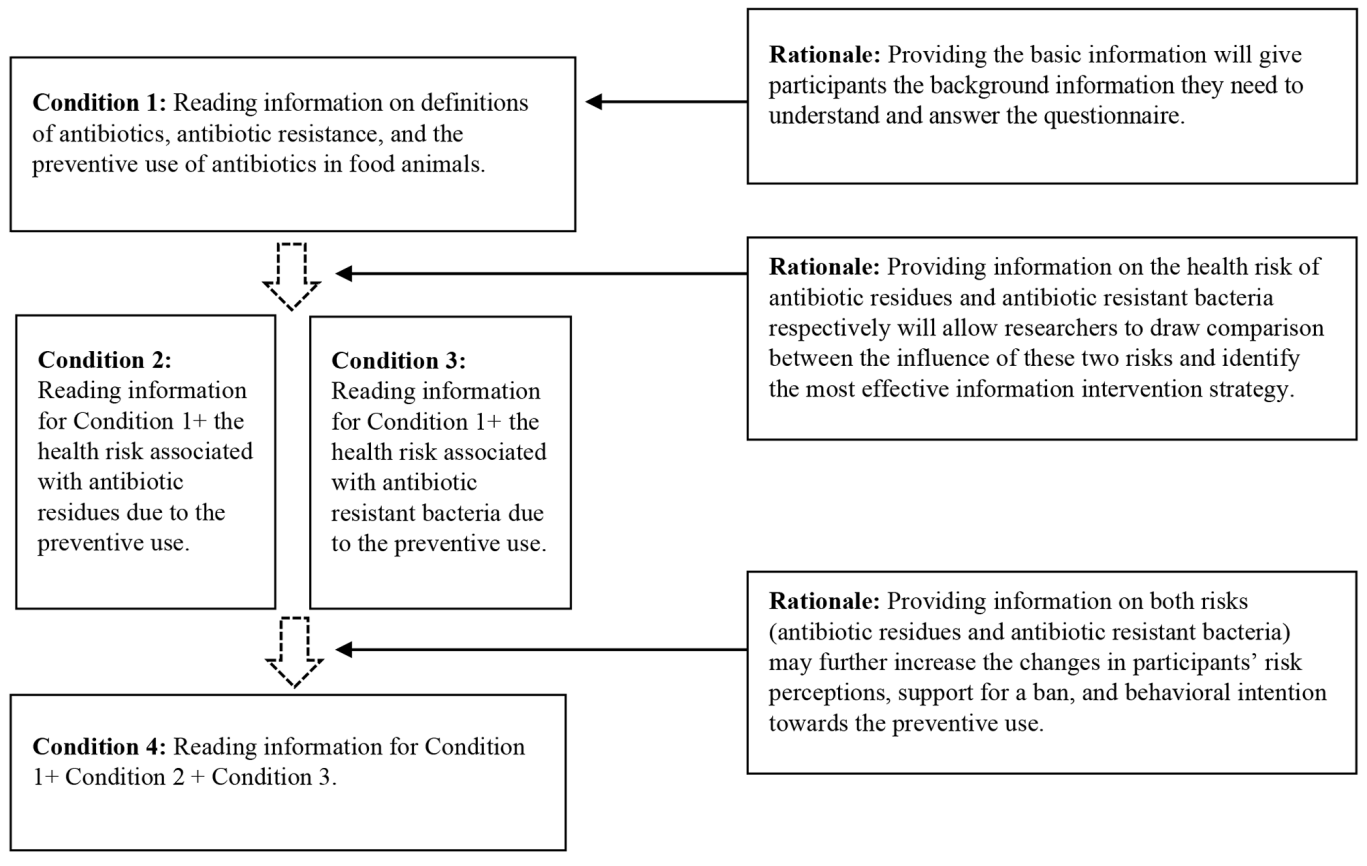
Experimental design diagram and rationale

An experimental study with four conditions was employed via an online survey in China. Condition 1 was the control condition, where participants read background information (i.e., definitions of antibiotics, antibiotic resistance, and the preventive use of antibiotics in food animals). Condition 2 further provided information on the effect of the preventive use on human health via increasing antibiotic residues in food, in addition to the information provided in Condition 1. Condition 3 provided information on the effect of the preventive use on human health via increasing the risk of getting infected with antibiotic resistant bacteria, in addition to the information provided in Condition 1. Condition 4 provided all information contained in the first three conditions. The provided information was developed based on findings in previous studies [1, 4, 6, 10, 11, 15, 19, 32, 44, 53, 85]. Figure 1 outlines the information provided for each condition and the underlying rationale. The experimental material is presented in Table 1.

**Table 1:** Experimental material provided for participants in each condition

Condition	Information
Condition 1	**Antibiotic and antibiotic resistance** • Antibiotics are used to treat bacterial infections in animals and humans. • Antibiotic resistance means bacteria, not animals or humans, become resistant to antibiotics. This makes antibiotics become less effective in treating certain bacterial infections. **The preventive use of antibiotics in food animals** • Antibiotics are often fed to healthy animals to prevent them from getting sick. For this purpose, antibiotics are often added to animal feed or water.
Condition 2	***Condition 1 plus the following*** **Toxic effects of antibiotic residues in food** • The preventive use of antibiotics in food animals may lead to antibiotic residues in food (e.g., meat, milk, and eggs), which may have direct toxic effects on humans after consumption. For example, high concentration of antibiotic residues may trigger allergic reactions in sensitive people and disrupt normal digestive function.
**Condition 3**	***Condition 1 plus the following*** **The preventive use of antibiotics in food animals may increase the risk of humans getting infected with antibiotic resistant bacteria** • Widespread use of preventative antibiotics in animal production increases the risk of new antibiotic resistant bacteria emerging. It may lead to the increased risk of antibiotic resistant bacteria being present in animals, in food, and in the environment (i.e., soil, river, groundwater, and seawater). This greatly increases the chance of humans being exposed to them. • People may get infected with antibiotic resistant bacteria when they have direct contact with animals, handle contaminated food, consume undercooked food, and have contact with antibiotic resistant bacteria in the environment. **What happens when humans get infected with antibiotic resistant bacteria** • If humans are infected with antibiotic resistant bacteria, it would be hard and sometimes impossible to treat because existing antibiotics are no longer effective in treating these bacterial infections.
**Condition 4**	**All information contained in the first three conditions.**

Participants were randomly assigned to one of the four conditions and were then asked to rate on a number of questions regarding risk perceptions (i.e., concern about antibiotic residues and antibiotic resistant bacteria, fear towards use of antibiotics as a preventative in food animals), support for a ban, and behavioral intention towards the preventive use (i.e., intention to buy meat produced without the preventive use of antibiotics).

### Procedure and participants

A professional online research platform (Credamo) was used to collect data. The survey link was sent to the participants panel of the research platform. Participants read the information and consent sheet first, which included a brief introduction to the study, information regarding participation and withdrawal (i.e., the participation is voluntary and participants can withdraw at any time), risks and benefits (i.e., no foreseeable risks), confidentiality (i.e., no personally identifiable information will be collected and all collected information will be treated confidentially), and contacts. Participants were asked to click ‘Next page’ button if they consent to take part in the survey. After answering the questions on demographics (i.e., gender, age group, and education), participants were randomly assigned to one of the four conditions, and then answered the questions on the dependent variables (i.e., risk perceptions, support for a ban, and behavioral intention regarding the preventive use). To ensure the participants would read the provided information carefully, timers were included. For participants in Conditions 1-4, the information was displayed on the page for 45, 60, 90, and 100 s respectively before being able to move on. The timers increased as the length of information increased, which was informed by the pretesting within the research team. A small fee was paid to participants who completed the survey.

A total of 2533 participants across China completed the survey. The majority of them were female (61.4%), at the age of 18 to 44 years (80.6%), and had completed at least a bachelor’s degree (80.6%). Table 2 presents participants’ demographic information for each condition.

**Table 2:** Demographics of participants

Variables	Condition 1 *N* = 625	Condition 2 *N* = 631	Condition 3 *N* = 638	Condition 4 *N* = 639
**Gender**				
Male	248 (39.7%)	231 (36.6%)	255 (40.0%)	245 (38.3%)
Female	377 (60.3%)	400 (63.4%)	383 (60.0%)	394 (61.7%)
**Age**				
18 - 24 years	150 (24.0%)	155 (24.6%)	143 (22.4%)	128 (20.0%)
25 - 34 years	232 (37.1%)	223 (35.3%)	217 (34.0%)	250 (39.1%)
35 - 44 years	125 (20.0%)	136 (21.6%)	149 (23.4%)	134 (21.0%)
45 - 54 years	69 (11.0%)	68 (10.8%)	80 (12.5%)	67 (10.5%)
55 + years	49 (7.8%)	49 (7.8%)	49 (7.7%)	60 (9.4%)
**Education**				
Senior high school and below (year 12)	58 (9.3%)	52 (8.2%)	42 (6.6%)	48 (7.5%)
College certificate	67 (10.7%)	77 (12.2%)	81 (12.7%)	66 (10.3%)
Bachelor’s degree	396 (63.4%)	397 (62.9%)	401 (62.9%)	405 (63.4%)
Postgraduate	104 (16.6%)	105 (16.6%)	114 (17.9%)	120 (18.8%)

### Measures

A 5-point Likert scale (1 = Strongly disagree, 5 = Strongly agree; unless stated otherwise) was provided for all responses. Cronbach’s alpha was calculated for multi-item measurements to examine the reliability of these measurements. An *α* value of 0.70 and above was considered acceptable [86, 87]. The average scores of items for multi-item measurements were used in data analysis.

Three aspects were measured to examine participants’ risk perceptions: concern about antibiotic residues, concern about antibiotic resistant bacteria, and fear towards use of antibiotics as a preventative in food animals. *Concern about antibiotic residues* was measured by asking participants to indicate their degree of agreement with four statements adapted from Michaelidou and Hassan [88] : “I think most meat contain antibiotic residues,” “I’m concerned about the amount of antibiotic residues in meat,” “Antibiotic residues are widespread in the environment,” “I’m concerned about the amount of antibiotic residues in the environment,” (*α* = 0.76). *Concern about antibiotic resistant bacteria* was measured by asking participants to indicate their degree of agreement with four statements adapted from Michaelidou and Hassan [88] : “I think most meat contain antibiotic resistant bacteria,” “I’m concerned about the amount of antibiotic resistant bacteria in meat,” “Antibiotic resistant bacteria are widespread in the environment,” “I’m concerned about the amount of antibiotic resistant bacteria in the environment,” (*α* = 0.77). *Fear towards use of antibiotics as a preventative in food animals* was measured by asking participants to express their feelings of fear (frightened, anxious, and worried) towards use of antibiotics as a preventative in food animals (1 = Not at all, 5 = Very much; α = 0.83) (Adapted from Milne et al. [89]).

*Supporting a ban for the preventive use* was measured with “Please indicate to what extent do you support a ban for the preventive use of antibiotics in food animals?” (1 = I don’t support at all, 5 = I totally support) (Adapted from Lusk et al. [90]).

*Intention to buy meat produced without the preventive use of antibiotics* was measured by asking participants to indicate their degree of agreement with two statements adapted from Bradford et al. [54]: “I intend to buy meat produced without the preventive use of antibiotics” and “I will look for meat produced without the preventive use of antibiotics” (*α* = 0.75).

### Data analysis

Data analysis was conducted by using SPSS version 22.0. One-way analysis of variance (ANOVA) was utilized to test the differences in demographics and the dependent variables between the four conditions. For variables where significant differences were found between the four conditions, Tukey (when equal variance assumption was satisfied) and Games-Howell (when equal variance assumption was not satisfied) post-hoc comparisons with bias-corrected and accelerated bootstrap estimation (1,000 samples) were carried out. A 95% confidence interval (CI) of the difference between means was used to determine whether the difference was significant. A 95% CI without zero indicates that the difference is statistically significant.

## Results

A series of one-way between-subjects ANOVA analyses were first conducted to examine the differences in demographics (i.e., gender, age, and education). The results suggested that there were no significant differences in these demographic variables among the four conditions: gender, *F* (3, 2529) = 0.63, *p* = 0.598; age, *F* (3, 2529) = 0.93, *p* = 0.428; education, *F* (3, 2529) = 0.95, *p* = 0.417. These results indicated that any differences in the dependent variables between the four conditions were very likely due to the differences in information provision.

**Fig. 2:**
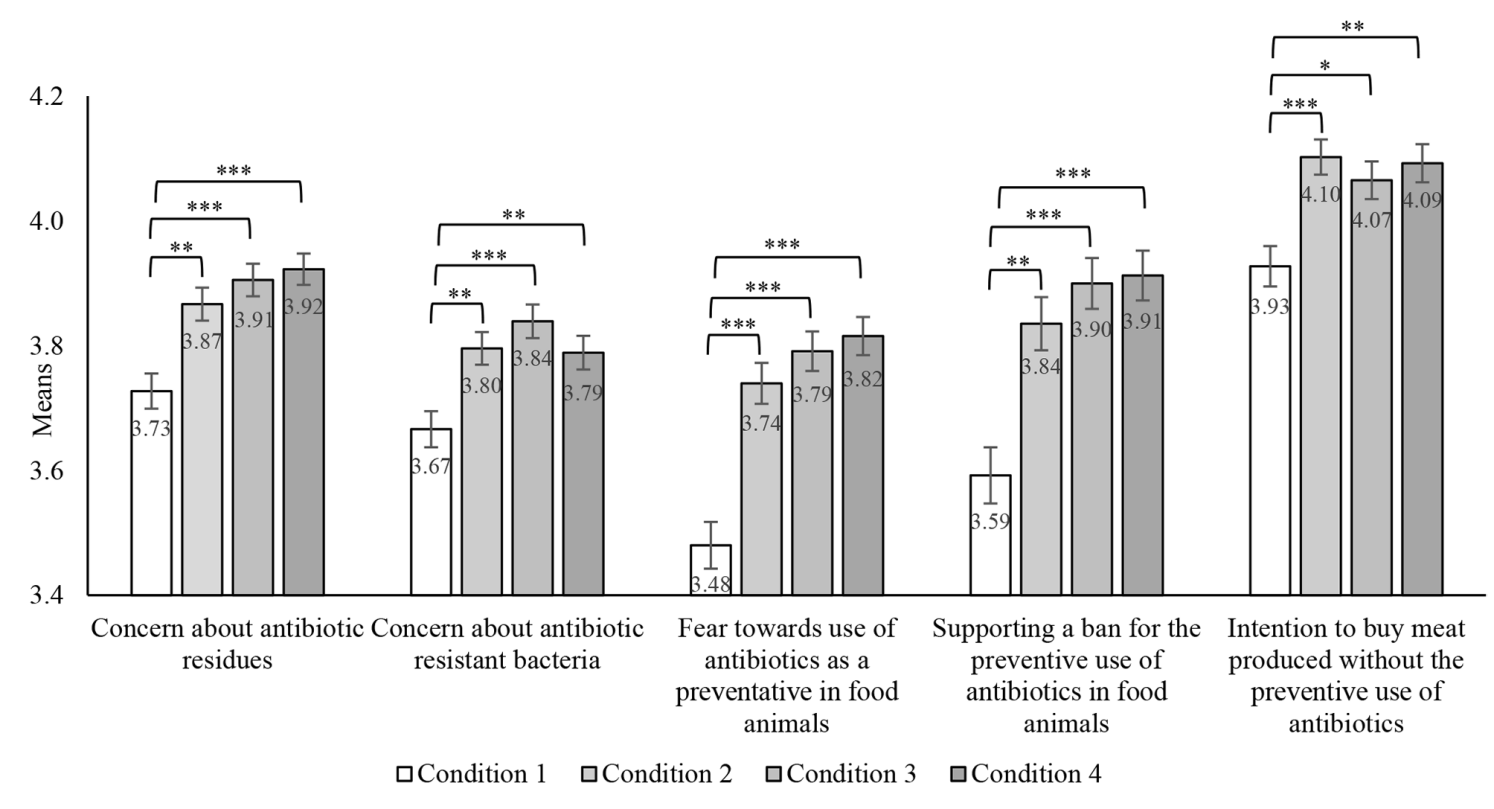
Means of the dependant variables with error bar. *Note* *p < 0.05, **p < 0.01, ***p < 0.001. Concern about antibiotic residues, concern about antibiotic resistant bacteria, and intention to buy meat produced without the preventive use of antibiotics were measured on a 5-point scale (1 = Strongly disagree, 5 = Strongly agree). Fear towards use of antibiotics as a preventative in food animals was measured on a 5-point scale (1 = Not at all, 5 = Very much). Supporting a ban for the preventive use of antibiotics in food animals was measured on a 5-point scale (1 = I don’t support at all, 5 = I totally support)

Another series of one-way ANOVA were carried out to further examine the differences in dependent variables (i.e., risk perceptions, support for a ban, and behavioral intention regarding the preventive use) across the four conditions. The results revealed significant differences among the four conditions in concern about antibiotic residues, *F* (3, 2529) = 11.11, *p* < 0.001, *η*_*p*_^*2*^= 0.013, concern about antibiotic resistant bacteria, *F* (3, 2529) = 7.38, *p* < 0.001, *η*_*p*_^*2*^= 0.009, fear towards use of antibiotics as a preventative in food animals, *F* (3, 2529) = 21.48, *p* < 0.001, *η*_*p*_^*2*^= 0.025, supporting a ban for the preventive use, *F* (3, 2529) = 12.47, *p* < 0.001, *η*_*p*_^*2*^= 0.015, and intention to buy meat produced without the preventive use of antibiotics, *F* (3, 2529) = 7.10, *p* < 0.001, *η*_*p*_^*2*^= 0.008. Post-hoc comparisons indicated that all these variables were significantly lower in Condition 1 than in all other conditions (Fig. 2), all *ps* < 0.05, all 95% CIs of the differences between means did not include 0. However, there were no significant differences in these variables between Conditions 2-4 (all *ps* > 0.05, all 95% CIs of the differences between means included 0). The descriptive statistics of dependent variables, the correlations between dependent variables and with demographics, and the 95% CIs of the differences between means of dependent variables in the four conditions are presented in Appendices A, B, and C, respectively.

## Discussion

The preventive use of antibiotics in food animals poses serious threats to human health globally. Previous research, however, suggested consumers have little knowledge about this practice. This study sought to examine the effect of information provision on the risk perceptions of, support for a ban, and behavioral intention towards the preventive use through an experimental study.

The results suggested that providing information on the health risks caused by the preventive use has significant influence on consumers’ risk perceptions, support for a ban, and behavioral intention regarding the preventive use. Compared to participants in control condition, where no health risk information was provided (Condition 1), participants who received information on the health risk of antibiotic residues (Condition 2), antibiotic resistant bacteria (Condition 3), and both antibiotic residues and antibiotic resistant bacteria (Condition 4) associated with the preventive use reported significantly higher level of risk perceptions of the preventive use (i.e., concern about antibiotic residues and antibiotic resistant bacteria, fear towards use of antibiotics as a preventative in food animals), stronger support for a ban on the preventive use, and a higher intention to buy meat produced without the preventive use. These findings demonstrated that increasing knowledge about the health risks of the preventive use was influential in increasing risk perceptions, support for a ban, and behavioral intention regarding the preventive use.

While there is no pre-existing literature allowing us to make assumptions about what differences it would make by providing participants with information on antibiotic residues or antibiotic resistant bacteria, this study revealed that information on the health risk of either antibiotic residues or antibiotic resistant bacteria led to similar levels of changes in risk perceptions, support for a ban, and behavioral intention in comparison to no health risk information being provided. That is, providing information on the health risk associated with either antibiotic residues or antibiotic resistant bacteria is equally effective in affecting these variables. Interestingly, when only information on the risk of antibiotic residues was provided, consumers’ concern about antibiotic resistant bacteria was also significantly increased, and vice versa. A possible explanation is that information on either of them could increase consumers’ overall risk perceptions of the preventive use. Therefore, providing information on the health risk of either antibiotic residues or antibiotic resistant bacteria enhanced the risk perceptions for both of them, despite that antibiotic residues and antibiotic resistant bacteria represented two different pathways in affecting human health. Besides, due to the abstract nature of antibiotic resistance, the understanding of this health threat was limited, resulting in misconceptions and low risk perceptions among the public [91–93]. Hence, it is likely that providing information about the transmission risks of either antibiotic residues or antibiotic resistant bacteria from animals to humans helped to make the issue of antibiotic resistance less abstract to the participants, thus increased the risk perception for both.

Furthermore, our findings suggested that providing information on both health risks in antibiotic residues and antibiotic resistant bacteria has a similar effect on consumers’ risk perceptions, support for a ban, and behavioral intention as providing information on only one of them. Although the pathways of how antibiotic residues and antibiotic resistant bacteria affect human health differ, the results demonstrated that providing information on both did not have an additive effect. This finding supported the hypothesis based on the plateau effect model rather than the linear model [78, 84]. That is, providing information on the health risk of either antibiotic residues or antibiotic resistant bacteria is effective enough to reach the plateau effect. Consequently, there is no significant additional effect by depicting both risks. Thus, consumers’ exposure to either information led to the greatest changes in risk perceptions, support for a ban, and behavioral intention.

The findings of the current study have important implications for livestock industries and policymakers. First, the findings provide insights for developing effective risk communication strategies to increase public risk perceptions and promote attitude and behavioral intention changes regarding the preventive use. Future risk communication can convey simple messages about the health risk of either antibiotic residues or antibiotic resistant bacteria associated with the preventive use, as exposure to information on either of them is influential. Noticeably, though the results of the present study indicated a “plateau effect” of information provision, it needs to be cautious when applying the findings to campaigns in the real world. Future research needs to further validate the results and explore if the “plateau effect” holds true in other scenarios. For instance, the information we provided on the health risks of both antibiotic residues and antibiotic resistant bacteria might be too long for the participants to fully process in the survey setting. Future research can provide information on both via shorter message and examine if there is additional effect. In addition, the effect of using video instead of text to convey the information should be examined, as video might be more influential than text [73]. Moreover, to make the health risks of antibiotic residues and antibiotic resistant bacteria more realistic and relevant to the public, future research can include storytelling from people who have been affected by antibiotic residues or antibiotic resistant infections [94]. Further, future studies can also explore the moderating variables between the relationship of information provision and consumers’ risk perception, support for a ban, and behavioral intention. For example, animal-derived food products produced without the preventive use of antibiotics might be more expensive than conventional products. Therefore, participants’ income level might be a moderator. Health literacy is also a potential moderator. It is the capacity to access, understand, evaluate, and use information to maintain or improve health and quality of life [95]. Low health literacy is related to less healthy choices and riskier behaviors [95]. It’s possible that information provision is more effective among people who have higher level of health literacy as they might be able to understand the provided information better and make a healthier purchase decision. Second, this study has important implications for antibiotic stewardship in food animals. The findings indicated that once the public is aware of the health risks posed by the preventive use, they express a stronger demand to ban the practice, which challenges the industries’ social license to operate. Such public demand will help facilitate the implementation of a ban on this practice. Further, this research highlights a potential market for animal-derived food produced without the preventive use of antibiotics among informed consumers, which could incentivize livestock industries to use antibiotics responsibly. This consumer preference is of great value for promoting responsible antibiotic use in food animals from One Health perspective, especially considering that the increasing concern about antibiotic overuse in food animals might lead to consumer demand for eliminating antibiotics in livestock industries [55, 56, 69]. Given the severe health risks posed by the inappropriate use and overuse of antibiotics in food animals, the public needs to be made more aware of the issue. However, people shouldn’t be alarmist and overzealously seek to boycott necessary use of antibiotics, as it is harmful to both human health and animal welfare.

While the current research shed lights on how building consumer knowledge can enhance consumers’ risk perceptions, support for a ban, and behavioral intention regarding the preventive use, there are some limitations. For instance, there might be some vegetarians among the participants. We did not filter out and exclude them because the proportion of vegetarians in Chinese population is relatively small (about 4-5%) [96] and the number of them should be reasonably balanced across the four conditions via random assignment. Future research should consider excluding vegetarians from analysis.

## Conclusions

To our best knowledge, this was the first study exploring the effect of information provision on risk perceptions, support for a ban, and behavioral intention regarding the preventive use of antibiotics in food animals. The findings demonstrated that providing information on the health risk of antibiotic residues, or antibiotic resistant bacteria, or both in relation to the preventive use is similarly effective in increasing consumers’ risk perceptions, support for a ban, and behavioral intention regarding this practice. These results suggested that increasing consumers’ knowledge about the health risk of either antibiotic residues or antibiotic resistant bacteria can lead to the greatest changes in these variables. The findings of the research can provide important insights to inform policymakers and livestock industries to develop effective communication strategies and public policies to promote responsible antibiotic use in food animals.

## Appendix A

Descriptive statistics of dependent variables (*M±SD*)

**Table:** 

	C1	C2	C3	C4	Total
Concern about antibiotic residues	3.73±0.71	3.87±0.67	3.91±0.66	3.92±0.63	3.86±0.67
Concern about antibiotic resistant bacteria	3.67±0.72	3.80±0.66	3.84±0.68	3.79±0.68	3.77±0.69
Fear towards use of antibiotics as a preventative in food animals	3.48±0.94	3.74±0.82	3.79±0.80	3.82±0.77	3.71±0.84
Supporting a ban for the preventive use of antibiotics in food animals	3.59±1.12	3.84±1.07	3.90±1.03	3.91±1.01	3.81±1.07
Intention to buy meat produced without the preventive use of antibiotics	3.93±0.81	4.10±0.71	4.07±0.76	4.09±0.77	4.05±0.77

*Note* C1, Condition 1, C2, Condition 2, C3, Condition 3, C4, Condition 4. Concern about antibiotic residues, concern about antibiotic resistant bacteria, and intention to buy meat produced without the preventive use of antibiotics were measured on a 5-point scale (1 = Strongly disagree, 5 = Strongly agree). Fear towards use of antibiotics as a preventative in food animals was measured on a 5-point scale (1 = Not at all, 5 = Very much). Supporting a ban for the preventive use of antibiotics in food animals was measured on a 5-point scale (1 = I don’t support at all, 5 = I totally support)

## Appendix B

Pearson correlations between dependent variables and with demographics

**Table:** 

		Gender	Age	Education	1	2	3	4
1. Concern about antibiotic residues	C1	0.06	0.07	0.01				
	C2	0.05	0.10*	-0.03				
	C3	-0.04	0.08	-0.07				
	C4	0.00	0.07	-0.04				
	Total	0.02	0.08***	-0.03				
2. Concern about antibiotic resistant bacteria	C1	0.06	0.08	-0.01	0.81***			
	C2	0.02	0.09*	-0.08*	0.78***			
	C3	0.01	0.04	-0.07	0.79***			
	C4	0.06	0.03	-0.07	0.78***			
	Total	0.04*	0.06**	-0.05**	0.79***			
3. Fear towards use of antibiotics as a preventative in food animals	C1	0.09*	0.12**	-0.04	0.64***	0.67***		
	C2	0.07	0.09*	-0.09*	0.55***	0.52***		
	C3	0.00	0.08*	-0.07	0.61***	0.58***		
	C4	0.03	0.06	-0.02	0.62***	0.59***		
	Total	0.05*	0.09***	-0.05*	0.61***	0.60***		
4. Supporting a ban for the preventive use of antibiotics in food animals	C1	0.01	0.17***	-0.01	0.24***	0.27***	0.34***	
	C2	-0.02	0.19***	-0.04	0.29***	0.31***	0.40***	
	C3	0.00	0.21***	-0.04	0.20***	0.20***	0.32***	
	C4	-0.03	0.09*	0.05	0.26***	0.22***	0.35***	
	Total	-0.01	0.17***	-0.01	0.26***	0.26***	0.37***	
5. Intention to buy meat produced without the preventive use of antibiotics	C1	-0.04	0.12**	0.02	0.30***	0.29***	0.34***	0.39***
	C2	-0.09*	0.19***	-0.03	0.21***	0.21***	0.29***	0.35***
	C3	-0.02	0.09*	0.00	0.29***	0.25***	0.28***	0.42***
	C4	-0.09*	0.13**	0.08	0.32***	0.29***	0.36***	0.39***
	Total	-0.05**	0.13***	0.02	0.29***	0.27***	0.33***	0.39***

*Note* C1, Condition 1, C2, Condition 2, C3, Condition 3, C4, Condition 4. *p < 0.05, **p < 0.01, ***p < 0.001. Gender, 1 = Male, 2 = Female. Age, 1 = 18-24 years, 2 = 25-34 years, 3 = 35-44 years, 4 = 45-54 years, 5 =55+ years. Education, 1 = Senior high school and below (year 12), 2 = College certificate, 3 = Bachelor’s degree, 4 = Postgraduate

## Appendix C

Differences in means of dependent variables between the four conditions

**Table:** 

		C1		C2		C3	
		*M*_*diff*_	95% CI	*M*_*diff*_	95% CI	*M*_*diff*_	95% CI
Concern about antibiotic residues	C2	-0.14**	(-0.22, -0.06)				
	C3	-0.18***	(-0.25, -0.10)	-0.04	(-0.11, 0.04)		
	C4	-0.20***	(-0.27, -0.12)	-0.06	(-0.13, 0.01)	-0.02	(-0.10, 0.05)
Concern about antibiotic resistant bacteria	C2	-0.13**	(-0.21, -0.05)				
	C3	-0.17***	(-0.25, -0.09)	-0.04	(-0.12, 0.04)		
	C4	-0.12**	(-0.21, -0.04)	0.01	(-0.07, 0.09)	0.05	(-0.03, 0.13)
Fear towards use of antibiotics as a preventative in food animals	C2	-0.26***	(-0.37, -0.16)				
	C3	-0.31***	(-0.40, -0.22)	-0.05	(-0.15, 0.03)		
	C4	-0.34***	(-0.44, -0.24)	-0.08	(-0.17, 0.01)	-0.02	(-0.12, 0.07)
Supporting a ban for the preventive use of antibiotics in food animals	C2	-0.24**	(-0.36, -0.12)				
	C3	-0.31***	(-0.42, -0.19)	-0.06	(-0.18, 0.05)		
	C4	-0.32***	(-0.44, -0.21)	-0.08	(-0.20, 0.03)	-0.01	(-0.14, 0.10)
Intention to buy meat produced without the preventive use of antibiotics	C2	-0.18***	(-0.26, -0.09)				
	C3	-0.14*	(-0.22, -0.05)	0.04	(-0.04, 0.12)		
	C4	-0.17**	(-0.25, -0.09)	0.01	(-0.06, 0.08)	-0.03	(-0.11, 0.05)

*Note* C1, Condition 1, C2, Condition 2, C3, Condition 3, C4, Condition 4. *M*_*diff*_, Differences in mean. 95% CI, 95% confidence interval. *p < 0.05, **p < 0.01, ***p < 0.001

## Data Availability

The datasets used in the current study are available from the corresponding author on reasonable request.
